# Emerging Time-Resolved X-Ray Diffraction Approaches for Protein Dynamics

**DOI:** 10.1146/annurev-biophys-111622-091155

**Published:** 2023-05-09

**Authors:** Doeke R. Hekstra

**Affiliations:** Department of Molecular and Cellular Biology and School of Engineering and Applied Sciences, Harvard University, Cambridge, Massachusetts, USA

**Keywords:** protein mechanics, serial femtosecond X-ray crystallography, time-resolved X-ray crystallography, electric field, excited states

## Abstract

Proteins guide the flows of information, energy, and matter that make life possible by accelerating transport and chemical reactions, by allosterically modulating these reactions, and by forming dynamic supramolecular assemblies. In these roles, conformational change underlies functional transitions. Time-resolved X-ray diffraction methods characterize these transitions either by directly triggering sequences of functionally important motions or, more broadly, by capturing the motions of which proteins are capable. To date, most successful have been experiments in which conformational change is triggered in light-dependent proteins. In this review, I emphasize emerging techniques that probe the dynamic basis of function in proteins lacking natively light-dependent transitions and speculate about extensions and further possibilities. In addition, I review how the weaker and more distributed signals in these data push the limits of the capabilities of analytical methods. Taken together, these new methods are beginning to establish a powerful paradigm for the study of the physics of protein function.

## INTRODUCTION

It has been a long-standing dream of scientists to directly observe proteins in action. In rare instances, it has been possible to do so by microscopy (84), and advances in high-speed atomic force microscopy now provide the first glimpses of protein dynamics in cell membranes (50). Progress also continues to be made on access to the excited states and kinetics of proteins via nuclear magnetic resonance (NMR) spectroscopy (109), and initial findings have been reported on how conformational heterogeneity observed in single-particle cryo-electron microscopy relate to protein dynamics at physiological temperature (11). Complementing these developments are dramatic advances in time-resolved X-ray diffraction (TRX), which now allows the visualization of protein dynamics over the full range of timescales from the fastest femtosecond-scale vibrations and chemical steps to millisecond- and second-scale conformational transitions in (near-)atomic detail.

In this survey of TRX, I address three overall questions: (*a*) How can we directly observe proteins in action? (*b*) How can we characterize the free energy landscape that enables these motions? Otherwise put, how do proteins respond to physical stimuli (since change in energy = applied force × induced displacement)? Finally, (*c*) how can we infer accurate models of protein excited-state conformations from TRX data and integrate these with existing computational models?

TRX excels when dynamics can be triggered by laser excitation of native chromophores, yielding amazing accomplishments like the direct observation of elementary steps in photosynthesis (58, 62, 67, 110). I only briefly cover progress on light-dependent proteins, since this work has been summarized in excellent reviews (16, 101). Instead, I focus on advances in TRX with a strong emphasis on areas where TRX is just beginning to demonstrate its potential: triggering progression along the reaction coordinate of enzymes and mapping the dynamical properties of proteins and enzymes more broadly. I focus on the present and future of the field; however, it should be noted that TRX builds not just on recent advances in source and detector technology, but also on heroic efforts during the 1980s and 1990s to create the field of time-resolved X-ray crystallography. In particular, Keith Moffat and his colleagues established many of the principles of the field, including the use of Laue diffraction (75), data collection modalities (10), and data processing (93), along with pioneering studies (49, 99) by others.

**Figure 1:**
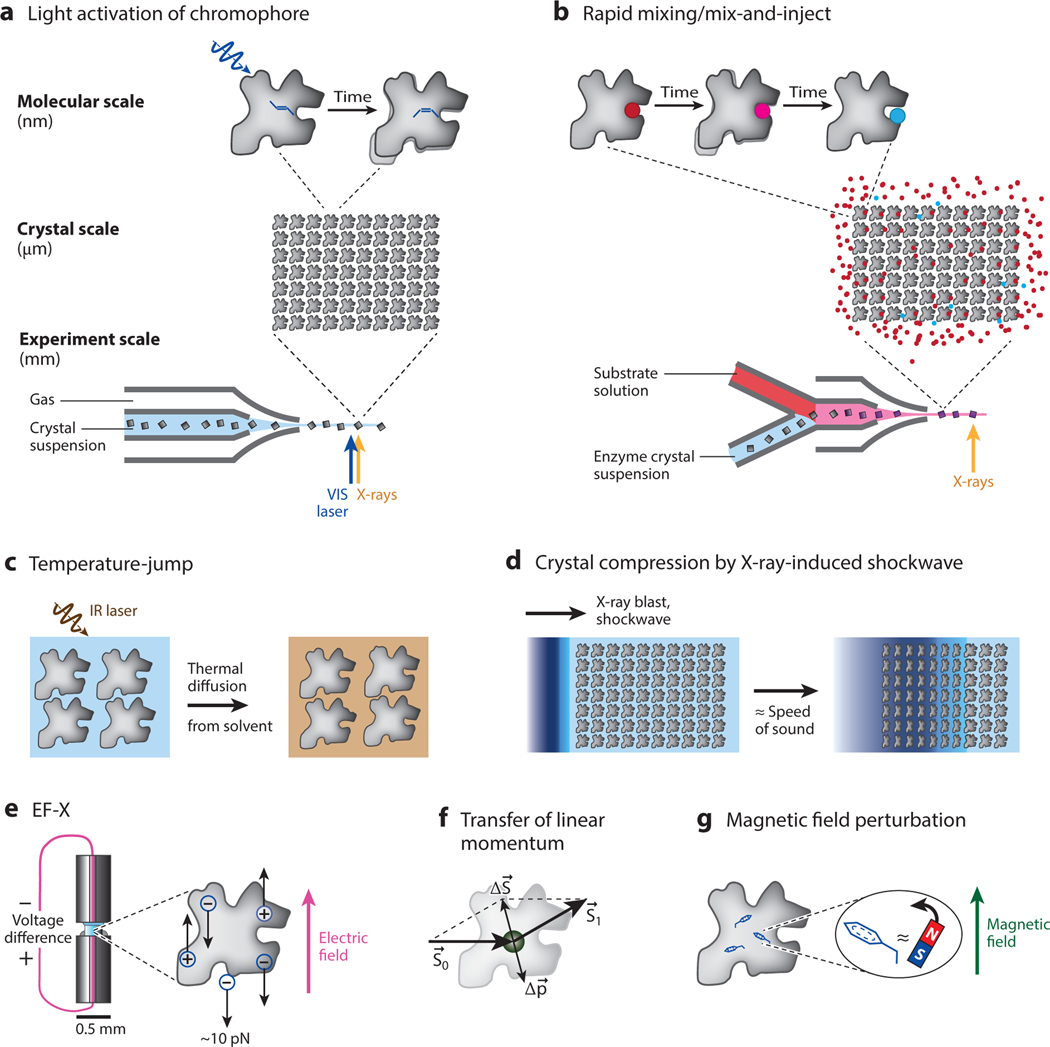
Perturbations for time-resolved X-ray crystallography. (*a*) In most time-resolved studies to date, laser light is used to drive a transition of a native chromophore (e.g., resulting in photoisomerization). As illustrated, such experiments are typically performed in a serial fashion, in which one X-ray diffraction image is collected per image. (*b*) In rapid-mixing serial crystallography, small crystals are rapidly mixed with ligands or substrates (*red spheres*) just before exposure to X-rays, e.g., resulting in enzyme products (*blue spheres*). Phenomena on timescales slower than the crystal diffusion timescale can be followed in this way. (*c*) In temperature-jump experiments, solvent is heated by an infrared laser pulse, and heat is transferred to the protein on the unit cell thermal diffusion timescale (<1 ns). (*d*) In X-ray pump, X-ray probe experiments, compressive shockwaves can be generated, rapidly compressing the protein molecules. (*e*) In EF-X experiments, an applied electric field directly exerts force on partial and elementary charges in proteins, resulting in a pattern of piconewton forces inducing conformational change. (*f*) X-rays, when scattered off atoms, impart linear momentum (Δ**p**). Differential acceleration of heavier atoms (larger *Z*) may be detectable in time-resolved experiments. (*g*) Ring currents in aromatic groups lead to an induced magnetic dipole moment in an applied magnetic field and therefore a torque toward alignment with the magnetic field. Currently achievable fields are likely too weak for this effect to be useful. Abbreviations: EF-X, electric-field-stimulated time-resolved X-ray crystallography; IR, infrared; VIS, light in the visible range (400–700-nm wavelength).

TRX was revitalized by the arrival of X-ray free electron lasers (XFELs) around 2009 and the first report on serial femtosecond X-ray crystallography (SFX) on a protein at the Linear Coherent Lightsource (LCLS) (25). This development followed an inspired proposal that protein structures could be determined from many microcrystals, or even single molecules, by the diffract-before-destroy principle (82)—a sample can diffract a number of X-ray photons far beyond the usual damage threshold if it does so before damage leads to significant displacement during the X-ray pulse (127). SFX has made many hard-to-crystallize targets newly tractable, leading, for example, to the determination of a multitude of membrane protein structures, including of many G protein–coupled receptors (108). A typical experimental design is shown in Figure 1a.

In parallel with the above-described advances in this field, there has been equally important progress in time-resolved X-ray solution scattering (2, 52, 68) driven by the same advances in instrumentation. Finally, traditional cryo-trapping experiments, in which progression through a series of states is quenched by rapid freezing, remain a valuable complement to TRX, yielding, for example, structures of intermediates in DNA replication (78), catalysis in dihydrofolate reductase (21), and the Kok cycle of photosystem II (PS II) (111).

## A BRIEF OVERVIEW OF DETECTORS, DELIVERY DEVICES, AND SOURCES

Tremendous advances in pulsed X-ray sources and detectors are enabling the growth of the TRX field. In particular, XFELs can now generate femtosecond-duration, extremely bright X-ray pulses at high pulse repetition rates. The advent of XFELs has driven rapid innovation in sample delivery platforms for serial crystallography, including gas virtual dynamic nozzles (32), electrospinning (105), tape drives (19, 38), acoustic droplet generation (96), microfluidic devices that generate segmented flows of precisely timed microdroplets (35), and chips that can hold tens of thousands of microcrystals (95). These devices are typically developed to improve crystal quality, hit rate, and sample consumption but are intriguing platforms for the development of new time-resolved diffraction experiments.

In addition, XFELs have driven the rapid development of new X-ray detectors to keep up with the increasing data rate and improve experimental accuracy. At (near-)continuous synchrotron X-ray sources, photon-counting detectors have achieved error-free detection of X-ray photons at significant count rates (<10^8^ photons per second per mm^2^; 23). With pulsed X-ray sources, however, many more photons may arrive per unit time during an exposure. New generations of integrating detectors, which measure total charge after a set exposure time, now enable TRX applications with similarly high sensitivity and frame rate. This includes CSPAD detectors (22) and, more recently, the ePix and JUNGFRAU integrating detectors, which have adaptive or multiple gain and single-photon sensitivity (77, 118).

## DIRECT OBSERVATION OF PROGRESS ALONG THE CONFORMATIONAL REACTION COORDINATE

The most direct way to learn how a protein works would be to observe its progression along the conformational reaction coordinate (CRC)—the sequence of conformational changes required for function. Time-resolved crystallography provides a fairly direct way of doing this with atomic and temporal detail—if progression along the CRC can be triggered, then TRX yields an ensemble-averaged movie of this progression. Key parameters to a successful TRX experiment are therefore (*a*) whether progression along the reaction coordinate can be triggered in a sufficiently large fraction of protein molecules, (*b*) with what level of synchrony one can do so, and (*c*) whether the process can be triggered repeatedly or is irreversible. Figure 1 illustrates some of the discussed techniques.

### Chromophore Excitation

The most successful way of achieving such synchronized progression along a CRC has been via absorption of light by a chromophore natively capable of triggering such progression (along with the dynamics of electronic degrees of freedom), as illustrated in Figure 1a. Many of the field’s most prominent examples fall into this category. In particular, photoactive yellow protein, a bacterial signaling protein, has been used not just as an interesting model system, but also as a paradigm for the establishment of new TRX experiments, including early synchrotron studies (42, 59, 94), TRX at XFELs (114), and MHz-rate TRX at EuXFEL (89). The series of electronic and conformational transitions, including the key trans-to-cis isomerization of the chromophore, is now well characterized; presumably, these transitions explain the physiological function of the chromophore (which is not as well characterized).

Myoglobin and hemoglobin are two other model systems (64, 65, 106, 107). Pioneering spectroscopic studies (5) used flash photolysis to dissociate ligands (CO or O_2_) from the heme group and established the rugged shape of the CRC of myoglobin with several metastable intermediates and the ensemble nature of the ground state. Early TRX measurements on myoglobin (106, 107) provided the structural correlates of these intermediates. Subsequent time-resolved solution scattering and SFX experiments showed that, on fast timescales (1–10 ps), the protein undergoes a damped ringing motion, suggesting strong coupling of the state of the heme group to collective vibrational degrees of freedom (6, 69).

More broadly, light-driven TRX is beginning to reveal the reaction coordinates of channelrhodopsins, which can pump or allow permeation of ions (85, 126) and are important optogenetics tools; of switchable fluorescent proteins (27, 125); and of phytochrome proteins, which mediate a host of sensing roles in bacteria and plants (26). The resulting insights into the sequence of conformational changes along the CRC may aid in engineering the specificity, efficiency, and kinetics of these proteins.

Finally, staggering progress has been made on understanding the mechanism of photosynthetic machinery, including PS II. PS II catalyzes the light-driven oxidation of water, liberating four electrons in four subsequent light-driven steps from two molecules of water and yielding molecular oxygen as a byproduct. A series of groundbreaking studies (62, 67, 110, 111) have made available atomistic models of metastable intermediates describing the structural dynamics of the oxygen-evolving complex (OEC); nearby waters acting as substrates and proton acceptors; quinone molecules, which accept electrons; and residues that mediate the requisite conformational changes of the OEC, quinones, and solvent motion. This work, the details of which are beyond the scope of this review, highlights several key aspects of TRX, including the interplay between scientific and technical advances—including crystal delivery systems such as the tape drive (19), the value of concomitant spectroscopic studies on crystals when these are possible, and the need for validation by difference electron density maps (98). As their authors intended, these studies are now inspiring synthetic photosynthetic systems (e.g., artificial leaves) intended to fulfill societal needs for energy and renewable resources (34).

### Alternative Direct Triggers of Functional Dynamics

The direct observation of light-driven functional protein motions is, of course, limited to the handful of proteins that respond directly to light. Their responses are also unusual: A visible-wavelength photon deposits about 100 *k*_*B*_*T* of energy into a protein within a few femtoseconds, mediated, initially, by electronic degrees of freedom. The conformational dynamics of most proteins are, instead, driven by much smaller forces. Two classes of TRX experiments stand out for their ability to directly trigger progression along a CRC despite the lack of native chromophores that can do so.

First, in photo-uncaging experiments, rapid formation of a molecule that triggers a functional transition is achieved by photolysis of a stabilized molecule to yield an active molecule. For example, in pioneering studies in the late 1980s, Schlichting and colleagues (99) obtained structures of hRas bound to GTP before hydrolysis. To do so, they photolyzed 2-nitrophenylethyl-GTP to yield GTP in the crystal. Photo-uncaging experiments have recently been reviewed in detail (76). Small molecules like H+ (photoacids), NO, CO_2_, and Zn2^+^ can be released by photoexcitation. Alternatively, conformational change could be triggered by interaction of proteins with photoisomerizable azobenzenes and stilbenes (15). These tools significantly extend the range of systems tractable by TRX.

Second, a yet more general approach is to flow in a substrate or cofactor to initiate a reaction or binding process and follow it with TRX. Ordinarily, there would be major obstacles to doing so in a crystal: As soon as the substrate binds to the enzyme and reacts to form the product, the show is over—one would need to do this just before an X-ray exposure, but dynamics will blur out on the timescale of a single enzymatic turnover. If one were to rapidly immerse a crystal in a bath of substrate, then the timescale of diffusion into a crystal would scale as the square of its thinnest dimension, which is slower than typical enzymatic turnover timescales even for thicknesses >10 micron. In other words, the substrate would reach the various active sites at moments in time spread all over the enzymatic cycle timescale, and crystallographically, one would merely observe an average of states.

Mix-and-inject (86, 100) and other rapid-mixing (19) serial crystallography approaches overcome both limitations by rapid mixing of microcrystals (5 microns or less in thickness) with concentrated substrate solutions, followed by probing with bright, single XFEL pulses by SFX. The approach is illustrated in Figure 1b. In this approach, a reaction is triggered only once in each crystal, and diffraction is obtained in a single shot before radiation damage has time to manifest. Mix-and-inject experiments have been demonstrated to provide insight into enzyme mechanisms for beta-lactamases (86), and their range of achievable timescales and enzymes will only increase.

Since protein crystals contain chemically and sterically heterogeneous solvent channels, diffusion timescales of small molecules in different crystal forms may vary by orders of magnitude, such that generic calculations have little relevance to specific systems. It therefore is crucial for design and interpretation of these experiments to calibrate diffusion timescales. Such calibration has been demonstrated by Pollack, Schmidt, and colleagues using both diffraction and EPR measurements (20, 90).

## GENERAL PROBES OF PROTEIN MECHANICS

What if we cannot drive transitions along the CRC? Alternatively, what if we want to understand how proteins enable progression along the CRC while precluding other motions? There are two main reasons to be interested in general physical perturbations that do not necessarily drive a protein along its reaction coordinate. First, it may be that it is impossible to directly trigger transitions along the CRC because of lack of a light-driven excitation strategy, substrate solubility, or diffusion constraints, or because the initial step along the CRC is so slow that it blurs out subsequent steps. Second, and more fundamentally, perturbative experiments can answer basic questions about the physical design of proteins: Which motions are possible beyond the CRC? How do the motions observed upon substrate binding, for example, relate to the intrinsic motions of an apo (free) enzyme? Can we reconstruct a plausible CRC by piecing together components from the repertoire of concerted motions of which each state is capable? How does the protein support conformational changes along a CRC while precluding nonproductive motions? Can we use such insights to re-engineer proteins, for example, to accommodate an alternative substrate or to implement the ability to allosterically modulate protein function with small molecules?

Traditionally, these questions have been addressed by methods that infer equilibrium conformational ensembles by NMR spectroscopy (12, 103, 109) or room-temperature crystallography (18, 36, 60, 117), usually combined with stabilization of states of interest using mutations or transition state analogs observed by conventional structure determination. Static physical perturbations have found limited application. These include changes in pH (120), temperature (37, 61), hydrostatic pressure (7), and humidity and osmotic pressure (4). TRX measurements of the dynamics triggered by rapid physical perturbations can, however, provide dynamic information and allow for strong perturbations that are intractable in their static form. Such measurements are just beginning to become feasible. In this section, I discuss recent developments and speculate on what may (not) lie across the horizon.

### Temperature-Jump Experiments

Wolff et al. (124) recently reported the first proof-of-concept temperature-jump (T-jump) TRX experiment using hen egg white lysozyme as a model system. In this experiment, a mid-infrared laser was used to heat microcrystals within a few nanoseconds, followed by SFX to observe the resulting conformational dynamics (Figure 1c). Importantly, general thermal equilibration on the length scale of unit cells takes place in less than a nanosecond ($\tau =L^{2}/D$ for a cell length of 100 Å, a thermal diffusion coefficient of approximately 1 × 10^−7^ m^2^/s, and a timescale of 1 ns). The timescale on which the protein relaxes its conformation is, however, dictated by its intrinsic physics—exactly the motions of interest. Interestingly, the diffuse scattering background observed in the diffraction image can serve as a reliable internal measurement of temperature.

Wolff et al. (124) describe a change in overall B-factors, which dominates the difference electron density map between the unperturbed crystals and the earliest time point (20 ns after the T-jump), a natural expectation given the increase in temperature (37). At longer time points (20 and 200 μs), however, the contribution of global disorder gives way to site-specific differences resulting from specific conformational changes. These changes are, moreover, sensitive to inhibitor binding, suggesting that large, slow conformational changes are coupled to the state of the active site and may be important for substrate binding and/or product release.

An exciting extension of T-jump experiments would be the ability to inject thermal energy site-specifically, e.g., using infrared-absorbing moieties in the silent region, or transparent window, of biomolecules around 1,800–2,500 cm^−1^ (1). Examples of such vibrational handles are alkyne and C-D bonds, which could be inserted as unnatural or deuterated amino acids. In particular, such probes would increase the value of studying timescales faster than 1 ns and could reveal the flow of thermal energy through a protein, analogous to thought-experiment simulations that use site-specific injection of thermal energy to reveal patterns of energy flow (54, 104).

Even without these future extensions, T-jump TRX experiments can now provide critical new data to build and refine physical models of protein dynamics and may serve as stringent benchmarks of molecular dynamics simulations.

### X-Ray Pump, X-Ray Probe Methods

X-ray pump, X-ray probe experiments are made possible by so-called split-and-delay (129) capabilities at XFEL facilities, generating pairs of X-ray pulses, as well as megahertz X-ray pulse repetition rates of next-generation XFELs (46). These capabilities offer pathways toward probing protein physics. X-rays can lead to reduction of metal ions and cysteine groups by the uptake of photoelectrons. Such photoreduction can trigger functionally relevant conformational transitions. For example, Gudmundson et al. (45) studied copper-dependent lytic polysaccharide monooxygenase. By collecting X-ray diffraction data from a single crystal while spreading out X-ray dose, the authors obtained structures of both the Cu(I) and Cu(II) states of the enzyme.

More recently, Nass et al. (80) reported time-resolved X-ray pump, X-ray probe experiments on lysozyme and thaumatin, observing clear increases in disulfide bond lengths in as little as 20 fs and continuing for at least 100 fs, an expansion at about the speed of sound but progressing more slowly and to a smaller extent than one would expect in a vacuum (80), indicating strong coupling to the surrounding protein and solvent matrix and to free photoelectrons. The results have repercussions for XFEL experiments with pulse lengths over 20 fs, as the observed diffraction will be affected by radiation damage. Because of the multifaceted nature of X-ray damage, including to aromatic and carboxylic amino acids, such X-ray pump, X-ray probe experiments may not constitute a viable way to probe physiologically relevant protein physics except in cases where change can be induced at sensitive metal ions or residues at a radiation dose that does not lead to widespread damage.

Intriguingly, XFEL X-ray pulses can also impulsively generate shockwaves in liquid jets commonly used for SFX (Figure 1d). These shockwaves propagate through liquid jets, including upstream crystals. This may pose a problem for high–repetition rate data collection at XFELs, as shockwaves can diminish diffraction quality and lead to structural perturbations. By clever use of a two-bunch mode at LCLS, Grünbein et al. (48) studied this problem with X-ray-pump, X-ray probe experiments on hemoglobin. The shockwaves appeared to result in pressure spikes on the nanosecond timescale, peaking in the 0.1–1 GPa range. This may result in strong uniaxial compression and possibly local pressure gradients steep enough to disrupt noncovalent interactions. The directionality and spatiotemporal dynamics of these shockwaves remain to be characterized further. It may be that shockwave properties can be tuned by varying jet viscosity and diameter, X-ray power, and crystal spacing. Regardless, it is exciting that neighboring crystals remained intact (with some loss of resolution) and exhibited apparent conformational changes. Since the exerted stresses are likely uniaxial (directional), it seems necessary to process this kind of TRX data in a lower-symmetry space group and sort by crystal orientation. Tools like those developed for post facto temporal positioning of diffraction images along a one-dimensional manifold (56) (e.g., the phase of the shockwave) provide a possible route toward extracting movies of proteins along the cycle of a propagating shockwave.

### Electric Field–Stimulated Time-Resolved X-Ray Crystallography

Electric fields provide a versatile tool to directly exert force on molecules through coupling of their electrical charges, q, with electric field, E, as F=qE. Proteins contain electrical charges. Full charges occur, for example, on carboxylate (−*e*) and amine (+*e*) groups, and partial charges, and therefore electrical dipoles, occur on peptide groups and water molecules. Nature itself uses electric fields to drive conformational change in voltage-sensing domains (113), to drive ions through ion channels (53), and to affect the function of other membrane proteins (8, 9). Response to local electric fields also mediates allosteric control by phosphorylation (91) and conformational change around RNA and DNA.

Exploiting this idea, Hekstra, White, Ranganathan, and colleagues (51) developed electricfield-stimulated time-resolved X-ray crystallography (EF-X). In an EF-X experiment, strong electric field pulses are applied to protein crystals, and the resulting motions are observed via short X-ray pulses (Figure 1e). Typical motions in proteins transport ∼1*e* of charge over ∼1 Å, requiring electric fields of ∼1 MV/cm to achieve energetic biases of ∼1 *k*_*B*_*T*. In terms of forces, 1 MV/cm corresponds to 108 N/C, or 16 piconewton per elementary charge—on the same scale as single-molecule force spectroscopy methods (81). To address the generality of EF-X as a method to study the physics of proteins, Hekstra et al. studied a PDZ domain, an abundant type of protein domain that does not require the ability to respond to an electric field for its function. They found that an electric field of 1 MV/cm was sufficient to cause pervasive conformational change, confirming that electric fields can generically probe accessible conformations. To examine the relationship between observed dynamics and protein function, they compared their results to the conformational changes observed in the PDZ domain family between apo and ligand-bound forms as a proxy for the conformational reaction coordinate and found substantial overlap between the motions observed by EF-X and the changes between apo and ligand-bound end points. By comparing their results to a high-resolution room-temperature crystallography data set (1.1 Å resolution), they further demonstrated that the induced excited states are detectable without the electric field but that the electric field can increase their occupancy sufficiently to enable determination of their structures.

At the time of this proof of concept, EF-X experiments failed on most crystals, even for the most robust crystal forms. The largest challenge has been the need for extensive manipulation of the crystals when placing them on electrodes, applying glue, and bringing in a second electrode. Through both mechanical stresses and exposure to air and liquid of slightly different osmotic pressures, translational order is readily compromised. Crystals with imperfect translational order (mosaicity) yield streaky diffraction spots under polychromatic X-ray exposure, which, with current detectors and software, leads to increased readout noise, poor geometric refinement, and spot overlaps. To address this, our group has developed new electrode devices and sample handling protocols (not yet published) that strongly reduce stresses on crystals and now enable data collection on delicate crystals.

As described above, existing sample delivery platforms may be modifiable to accommodate TRX experiments, and this can include the use of graphene as X-ray transparent electrodes (112) or microfluidic devices with integrated electrodes (63). These devices make the development of serial EF-X conceivable. A key consideration is the effective electric circuit of the experiment. In the experiments described by Hekstra et al. (51), a conductive path exists from electrode, through crystal and liquid, to electrode. A different class of designs, called capacitive designs, has been proposed several times informally. In such designs, a crystal is sandwiched between two dielectric layers (e.g., plastic or air) that act as capacitors. For resistors in series, the largest resistor will see the largest voltage drop, while for capacitors in series, the smallest capacitor will see the largest voltage drop in steady state. Protein crystals are wet and will, in general, support both capacitive and resistive current. Capacitive designs, therefore, have a maximal timescale beyond which resistive sample currents will drain any remaining voltage drop over a protein crystal. This timescale depends sensitively on physical details and is best characterized experimentally.

It is also conceivable to use the electric field component of light to exert force on atoms, as light propagation is accompanied by orthogonal time-varying electric and magnetic fields. Specifically, single- or few-cycle pulsed lasers of THz or higher frequency can generate transient pulses with electric field strengths at or above the 1 MV/cm range (39, 128). These field strengths are comparable to those in EF-X experiments. The energetic biases that can be attained at 1 MV/cm field strength are modest for low-amplitude picosecond collective vibrations (amplitudes typically below 1 Å), but such experiments are now, in principle, possible. Analogous to capacitive designs, however, the relative permittivity of water sets an upper bound on the timescales on which such experiments can be performed: Water molecules can reorient on the single-picosecond timescale and therefore screen out most of the electric field. Therefore, the effective field strength experienced in crystals during a passing electromagnetic pulse will depend strongly on experimental conditions and pulse frequency.

Despite some challenges, there is good reason for optimism: A permanent setup for EF-X is available at the BioCARS facility at the Advanced Photon Source, including a pulse generator that can generate pulses with positive and negative half-waves with full control of pulse parameters; new devices are improving success rates; samples can now be prepared off-site; and progress on the data analysis pipeline, including for scaling and merging of polychromatic diffraction data (see below), is beginning to relieve demands on diffraction quality.

### Other Possibilities: Photon Momentum, Quantum Mechanics, and Magnets

One could imagine other ways of physically perturbing protein molecules. For instance, photons carry a linear momentum p=b/λ, where b is Planck’s constant, and λ is the wavelength, which can be used to move atoms and objects. This is the idea behind, for example, solar sails for space exploration. Photon momentum can be transferred to atoms during elastic (Thomson) scattering or to electrons, such as in Compton scattering. Since Compton recoil electrons and photoelectrons travel some distance (97), it appears that Thomson scattering provides the only means to impart momentum to specific atoms (Figure 1f). The momentum imparted depends on scattering angle but on average will be aligned with, and of similar size to, the photon’s momentum. To get to photons with a momentum comparable to thermal momenta, one needs X-rays. For example, a 1 Å X-ray photon carries *p* 6.6 10^−24^ kg m/s, while a typical atom of mass *m* of about 2*Z* Dalton has a root-mean-square momentum due to thermal fluctuations $p_{rms}=\sqrt{mk_{B}T}\sim 2.5\times 10^{-24}\sqrt{Z}$ kg m/s for atomic number Z. Since X-ray absorption is Z and edge dependent, there may well exist a spectral and chemical window in which site-specific Thomson scattering results in displacements visible by X-ray pump, X-ray probe experiments at X-ray doses that do not obliterate crystals immediately. Just as in EF-X (51), however, the deposition of linear momentum is a vectorial perturbation. It is therefore essential to analyze the resulting diffraction data in the appropriate reduced-symmetry space group (most generally P1) and to account for the orientation of each crystal relative to the X-ray beam when doing so.

Recently, it has also been proposed that radiation may directly stimulate certain quantum phenomena. For example, Katona and colleagues (70) described the putative observation of quantum behavior in the collective vibrational modes of hen egg white lysozyme crystals stimulated by low-amplitude terahertz laser pulses. Without a doubt, the quantum-mechanical nature of matter can manifest itself in surprising ways, and this research direction deserves further elaboration.

Finally, magnetic fields could, conceivably, provide another way to directly interact with proteins—in particular, when a magnetic dipole has a structural rather than electronic basis. To assess the feasibility of this idea, I looked at the clearest example of such a structural magnetic dipole, that of aromatic ring currents (Figure 1g). Based on existing measurements (28, 30), the typical magnetic susceptibility of an aromatic ring is approximately 10^−4^ cgs emu, corresponding to around 10^−9^ m^3^/mol in SI units. The corresponding energy of an induced magnetic dipole in a magnetic field comes to 0.3 J/mol at 20 T or approximately 8 J/mol at 100 T, among the largest static and pulsed magnetic field strengths, respectively, that are currently achievable. For comparison, applying Ampère’s law, at a 5 kA peak current in an LCLS free electron laser pulse, one obtains a transient magnetic field of only 1 T at 1 mm from the electron beam. Under none of these conditions is the energetic bias close to 1 *RT* (2.5 kJ/mol). In other words, such experiments are likely to remain beyond the horizon for the near future, but I am happy to be proven wrong!

## GROWING PAINS: ANALYSIS OF TIME-RESOLVED X-RAY CRYSTALLOGRAPHY EXPERIMENTS

Time-resolved X-ray diffraction has inherited much of its analytical machinery from conventional X-ray crystallography, but analysis of time-resolved diffraction data poses its own challenges. In this section, I briefly discuss (*a*) challenges inherent in the use of short X-ray pulses, (*b*) challenges in accurately extracting the often subtle changes in structure factor amplitudes, (*c*) challenges inherent in obtaining refined models of excited states when only a small fraction of molecules is actually perturbed away from the ground state ensemble, and (*d*) challenges in combining insights from time-resolved X-ray diffraction experiments and simulations of protein dynamics. Each of these sets of challenges is formidable and needs to be addressed to make the recording of molecular movies and the building of effective physical models of proteins routinely possible.

### Partiality and Polychromatic Time-Resolved X-Ray Diffraction

In a time-resolved experiment, protein crystals are illuminated using picosecond or femtosecond X-ray pulses. This precludes use of the conventional rotation method, in which crystals are rotated during exposure to fully observe the intensities of the Bragg reflections. As a result, individual XFEL time-resolved observations are always partials (but see 119 for a quasirotation experiment at an XFEL), and additional information is needed to get from partial observations of reflection intensities to merged structure factor amplitudes. The implied extrapolation can be done based on parametric models of the shape of reflections in reciprocal space, e.g., using a Lorentz or Gaussian line shape (44, 116); be based on a model of lattice disorder and forward modeling (66, 74); or be implied by a neural network (29). These methods rely on accurate experimental geometry and can improve the accuracy of estimated structure factor amplitudes.

To generate sufficiently bright X-ray pulses for TRX, synchrotron-based sources typically use a beam spectral bandwidth of 1–5%, in contrast to monochromatic radiation (0.01% bandwidth or less) and XFEL SASE spectra (self-amplified spontaneous emission; approximately 0.3% bandwidth). Historically, the main reason for using these pink or Laue pulses was the increase in photon flux that comes with keeping more of the photons generated by undulators (47). A second advantage is that, when pink X-ray sources are used, most reflections are full reflections: Slightly different wavelengths will interfere constructively from unit cells of slightly different orientation, altogether capturing the full diffraction intensity. By the same logic, more reflections can be observed per diffraction image. The benefit of such pink sources for serial crystallography was recently demonstrated at both a synchrotron source [BioCARS (72)] and an XFEL [SwissFEL (79)]; in both cases, there were large reductions in the required numbers of diffraction images over data collection using other sources.

A major drawback of pink radiation has been the limited availability of software capable of indexing polychromatic diffraction images, resolving harmonic overlaps (reflections of which of the Miller index triplets are rational multiples of each other), and correcting for the wavelength dependence of constructive interference and absorption. The first step, indexing, was recently addressed in new open-source software, PinkIndexer (43), which interfaces with CrystFEL (121), and the latter two steps have been addressed in Careless (29). Efforts are underway to support the steps preceding scaling and merging in the DIALS framework (122). In other words, long-standing challenges in analyzing pink diffraction may soon be mitigated sufficiently for pink diffraction to become an attractive modality for TRX at synchrotrons and XFELs alike. One final issue is the inevitable radial streaking commonly observed when mosaic crystals are exposed with a pink beam (14). This remains an obstacle for several reasons: With radial streaking, standard analysis methods that identify spot centroids and integrate counts over an integration mask become less dependable, more pixels mean larger readout noise, and spots more often overlap. Forward modeling, in which the counts at each pixel are repeatedly predicted based on intermediate estimates of crystal properties and experimental geometry (74), are computationally intensive but may ultimately make pink beam data analysis tractable even for somewhat mosaic crystals. The most recent detectors, moreover, mitigate the increase in readout error for streaky diffraction data.

### Extracting Small Differences

The structural changes observed in TRX are often modest in terms of their effect on overall structure factor amplitudes. Moreover, in most TRX experiments, a large portion of the molecules remain in the ground-state ensemble—in light-stimulated experiments, this is because chromophores have limited quantum efficiency or exhibit multiple relaxation pathways, and in other TRX experiments, it is because the perturbation may be much less strong or the response may be blurred out over time (e.g., due to rate-limiting diffusion of substrate). Therefore, the identification of excited states of a protein is often much more challenging than the identification of the ground state. The needs of TRX are not well supported by crystallographic analysis suites like PHENIX and CCP4.

Furthermore, while observed intensities are proportional to the squared structure factor amplitudes, the proportionality factor is not a constant. As discussed above, diffraction intensity can vary with distance of reflections from a perfect diffraction condition (the Ewald offset) and with the wavelength at which constructive interference takes place and can be modulated by crystal mosaicity and size. In addition, beam polarization, detector geometry [angles subtended, absorption, point-spread function, (mis)calibration], and radiation damage affect diffraction intensity (55). Therefore, care is required to put the observed intensities on the same scale (scaling) before extracting merged structure factor amplitudes. Several approaches have been developed to address scaling imperfections, but room for improvement remains. For example, after scaling, it is commonly observed that ON (perturbed) and OFF structure factor amplitudes still show systematic differences in magnitude—this can be due to both real effects (perturbed crystals often exhibit increased disorder and therefore decreased amplitudes) and systematic errors in scaling. These scale differences are often addressed by after-the-fact scaling of one time point to another using SCALEIT (57) (see, e.g., 85, 114, 123); by local scaling in SOLVE (51, 71); or, for difference maps, by PHENIX’s isomorphous difference map routine, which appears to be undocumented (e.g., 21, 83). Time points can sometimes be scaled jointly and merged separately [e.g., in CrystFEL (114) or Careless (29)].

Fundamentally, again, scaling poses a dual estimation problem: to infer both the scales of reflections and the merged structure factor amplitudes. Although different time points or conditions in TRX experiments should not yield identical structure factor amplitudes, they should often be close. Exploiting this similarity may be key to mitigating the tradeoff between scaling accuracy and coverage of the time domain.

Specifically, even though protein dynamics are intrinsically high dimensional, average electron density dynamics follow single-dimensional trajectories (in a high-dimensional space). TRX studies can therefore, in principle, be effective with a much smaller number of diffraction images per time point than in current practice (56, 102). Two approaches already exploit continuity between time points to improve inference of changes in structure factor amplitudes. First, Schmidt, Moffatt, and colleagues (102) developed singular value decomposition to infer the structures of intermediates in the time evolution of PYP, for which a mixture of intermediates is present at many time points, achieving both a separation of states and averaging of signal present at different time points. A second, more recent advance is the use of machine learning to sort diffraction images along an underlying one- or low-dimensional manifold (56). This approach has proved powerful in the analysis of ultrafast dynamics for which the estimated time stamps are inaccurate relative to the timescale of meaningful variation in average dynamics, as illustrated vividly for dynamics associated with the crossing of a conical intersection in PYP with concomitant abrupt changes in electron density (56). Intriguingly, this latter approach is embedded earlier in data processing, possibly improving scaling and postrefinement beyond what is achievable with post hoc corrections.

More generally, the basic elements exist to exploit the correlations between time points and conditions in a statistically efficient manner. This includes Bayesian formalisms (17, 29, 40), multivariate priors (92), machine learning algorithms, and large computational power.

### Refining Excited State Models

The next goal of TRX is the elucidation of the structures of the excited states of proteins. These excited states are typically present at low occupancy, and conventional refinement algorithms will often simply return the ground state when performing naive refinement against perturbed (ON) data. To refine structural models, one could extrapolate from the observed difference in structure factor amplitude (e.g., $\left|F_{\text{on}}\right|-\left|F_{\text{off}}\right|$) and the ground state $\left(|F_{\text{off}}|\right)$ to something more akin to the structure factor amplitudes of the excited state, e.g., as $\left|F_{\text{ext}}\right|=N\left(\left|F_{\text{on}}\right|-\left|F_{\text{off}}\right|\right)+\left|F_{\text{off}}\right|$. This calculation of so-called extrapolated structure factor amplitudes (ESFAs) was first introduced by Genick, Srajer, Moffatt, and colleagues in 1997 (41, 42) and remains widespread practice.

There is substantial variation in the field, however, in how ESFAs are calculated, e.g., whether to use the calculated or observed $\left|F_{\text{off}}\right|$ in the second term; whether to apply weights; and how to calculate the extrapolation factor based on, for example, the appearance of negative electron density features (126) or optimization of the $\left|F_{\text{ext}}|–|F_{\text{off}}\right|$ difference map, e.g., around a chromophore (26, 125). For photoexcitation studies, the extrapolation factor can be directly interpreted in terms of excitation efficiency, while for more distributed perturbations, extrapolation simply amplifies the contribution of excited states to the electron density (while also amplifying errors). Calculation and analysis of ESFAs is still largely based on per-group custom scripts, but a tool may soon be available that brings together different forms of ESFA calculation (31).

Extrapolation of structure factor amplitudes is based on an obvious approximation—that the phases of the ground state and ON state (mixed ground and excited state) do not differ significantly. One approach to loosening this assumption is to re-extrapolate once structure refinement provides information about the phase differences between ground and excited state (101, 115, 126), to do so iteratively (87), or to use putatively representative structures to provide phase differences for extrapolation (26). Validation of this approach, e.g., using synthetic data, is necessary to understand its convergence properties and sensitivity to model errors. Since the sign of the phase difference between ground- and excited-state structure factors does not affect the extrapolation, model bias seems to be a limited risk.

A further step toward accurate extrapolation is the refinement of excited states against ON data in the presence of a fixed ground-state model. This approach implicitly considers the phase difference between ground state and excited states at each step, adjusting the implied extrapolation continuously during refinement. Neutze and colleagues (33) successfully applied a limited variant of this protocol (with fixed occupancy and applied to a limited region). This approach could be combined with restraints on the real or reciprocal space differences between ground states and excited states and/or exploit similarities along time series to finally yield a common platform for automated refinement of excited states that can achieve the movies that the field painstakingly strives for.

### Integration with Computational Methods

TRX provides experimental trajectories of the electron density of proteins in (nearly) atomic detail across a wide range of timescales (16). At the same time, molecular dynamics (MD) simulations allow replication of trajectories of the dynamics of individual protein molecules across the same timescales, which allows an observer to know the forces and energies leading to the conformations and kinetics observed. TRX data are inherently ensemble measurements, which can obscure and smooth out transitions (especially when a fast transition happens following a slow one), and the inference of atomistic movies is further hampered by errors in extrapolated structure factor amplitudes. MD simulations, in contrast, attain detailed atomistic trajectories, but these are subject to systematic errors in energetics and, therefore, errors in kinetics and systematic conformational biases. Attaining sufficient sampling of conformational space often remains a challenge as well.

Despite the complementary strengths of MD simulations and TRX experiments, advances at their interface are slow. MD simulations may be used to motivate feasibility in beamtime proposals, but in the literature they are, at best, used for qualitative comparisons (e.g., 3, 33, 83) (confusingly, figures displaying calculated difference electron density maps usually refer to the difference in electron density calculated for structural models refined against different time points, rather than to results from MD simulations). More rigorous comparisons between MD and crystallographic experiments are found elsewhere, e.g., in comparison to protein diffuse X-ray diffraction measurements (73). Part of the difficulty stems from systematic errors in forcefields and/or system preparation (e.g., of the disordered solvent component; 24), leading to systematic displacement of molecules relative to crystal structures, which degrades the ability to directly compare structure factor amplitudes or electron density. Attempts at quantitative comparison have been limited due to multiple causes: a lack of interaction between the TRX and MD fields; a focus by the MD community on quantities (atomic coordinates) that are not primary crystallographic observables (structure factor amplitudes or electron density maps); and a credit assignment problem—when MD predictions do not match experimental observations, what does one change? Forcefields contain thousands of different parameters that contribute in myriad ways to the simulation outcome. Solutions could include developing protocols for maximum entropy modification of forcefields (13) or simplifying the physical models used, e.g., to Markov state models (88), such that fine-tuning on the basis of experiment becomes unambiguous.

## CONCLUSION

In this review, I describe key developments in time-resolved X-ray diffraction studies on proteins, with an emphasis on techniques that do not rely on the ability to trigger progress along the reaction coordinate based on a native chromophore. These approaches still span the range from nearly inconceivable (e.g., pulsed magnetic field studies) to nearly mature (e.g., some photo-uncaging and rapid-mixing studies). Overshadowed by technical and instrumentation advances, analytical challenges are receiving too little attention; solving them may enable more efficient use of scarce pulsed X rays.

Much of the terrain of TRX, then, remains poorly charted, and the limits of what is possible remain unclear. Nevertheless, the terrain promises to provide paths toward the understanding of the physical basis of function for a wide array of biomolecules. The hoped-for reward is not just the ability to piece together the conformational reaction coordinate of proteins, but also a deeper understanding, in the form of experimentally constrained physical models, of how proteins shape their functional transitions while closing off a multitude of unproductive transformations. Such insight, in turn, may tell us how nature designed its myriad miraculous machines and how to design some of our own.
